# Trends in prevalence and treatment of antepartum and postpartum depression in the United States: Data from the national health and nutrition examination survey (NHANES) 2007 to 2018

**DOI:** 10.1371/journal.pone.0322536

**Published:** 2025-04-30

**Authors:** Rebecca M. Gardner, Pervez Sultan, Rebecca A. Bernert, Julia F. Simard

**Affiliations:** 1 Department of Epidemiology and Population Health, Stanford University, Stanford, California, United States of America; 2 Department of Anesthesiology, Perioperative and Pain Medicine, Stanford University, Stanford, California, United States of America; 3 Department of Psychiatry and Behavioral Sciences, Stanford University, Stanford, California, United States of America; 4 Stanford Medicine, Stanford Health Care, Stanford, California, United States of America; 5 Division of Immunology and Rheumatology, Department of Medicine, Stanford School of Medicine, Stanford, California, United States of America; 6 Department of Obstetrics and Gynecology, Stanford School of Medicine, Stanford, California, United States of America; Neurocrine Biosciences Inc, UNITED STATES OF AMERICA

## Abstract

**Objectives:**

(1) To assess the prevalence of depression and treatment rates in antepartum and postpartum women compared to a control group of reproductive-age women over a 12-year period, and (2) To determine demographic characteristics associated with depression.

**Methods:**

National Health and Nutrition Examination Survey data (2007–2018) were used. 5412 controls, 314 antepartum women, and 455 postpartum women were analyzed. Outcomes included depression prevalence, defined as moderate to severe depressive symptoms measured by Patient Health Questionnaire-9 (PHQ-9) scores ≥10 or self-reported antidepressant use; and treatment, defined as antidepressant prescription and/or mental health care services in the past 12 months. Multivariable logistic regression adjusted for age, insurance, race/ethnicity, education, and marital status estimated odds ratios and 95% confidence intervals.

**Results:**

Depression prevalence was 20.2% in controls (95% CI 18.5–21.9), 9.7% in antepartum (6.3–14.1), and 12.8% in postpartum women (9.3–17.1). Mental health care service utilization increased for postpartum women in 2017–2018 (22.0%, 10.6–37.7). In those with depression, control and postpartum groups had similar treatment rates (70%, p = 0.894) compared to antepartum women (51%, p = 0.051). Antidepressant use was the most common treatment reported in all groups. Those who were married or had private insurance had the lowest depression rates in their respective categories. After adjusting for confounders, antepartum and postpartum women had lower odds of depression compared to controls. When the outcome was PHQ-9 ≥ 10 alone, these associations persisted.

**Conclusion:**

In a nationally representative sample, depression prevalence was lower in perinatal women compared to reproductive-age controls, and treatment rates were lowest in antepartum women with prevalent depression. Mental health care services may have increased for postpartum women due to the US Preventive Services Task Force 2016 recommendations, which endorsed psychotherapy for postpartum women. Even so, antidepressants were the most reported treatment among perinatal women, despite psychotherapy being the first-line recommended treatment for this population.

## Introduction

Postpartum depression is the most common pregnancy-related complication worldwide and can lead to adverse maternal and child outcomes, including compromised mother-infant bonding, poorer infant cognitive outcomes, and shorter breastfeeding duration [1–4]. Likewise, antepartum depression is highly prevalent and tied to adverse birth outcomes, including preterm birth and low birthweight [5,6]. Both antepartum and postpartum depression are associated with increased risk for infant mortality and maternal suicide, which represents a leading cause of death in the perinatal period---prioritized nationally for prevention [7–10].

Depression prevalence has recently increased in the United States (US), largely driven by an increase in the incidence of depression in adolescents and young adults [11–13]. Globally, women demonstrate a two-fold higher risk of developing depression in their lifetime compared to men, and this gender gap has stayed constant over the last forty years [14,15], despite significant efforts to advance awareness and treatment.

Recent studies have documented an increase in depression and suicidal ideation in antepartum women in the US over the past two decades [16,17]. However, these findings are derived from analyses of commercial claims databases, which notably exclude mothers with public insurance. To our knowledge, no recent studies have assessed trends in the prevalence and treatment of antepartum or postpartum depression in a nationally representative US sample.

To address this gap in the literature, we analyzed trends in antepartum and postpartum depression prevalence and treatment compared to women of reproductive age, who were neither pregnant nor postpartum. We investigated trends over time and by key demographic characteristics known to be associated with risk and observed prevalence, including race and ethnicity, marital status, and education level. We harnessed data from 2007–2018 in the National Health and Nutrition Examination Survey (NHANES), a nationally representative and diverse population of the US conducted by the Centers for Disease Control and Prevention (CDC).

## Methods

### Study population

The study population included women of reproductive age between 20 and 44 years old, who underwent a urine pregnancy test, and were asked about their reproductive history and mental health from six NHANES cycles from 2007 to 2018 (each cycle comprises two years of data). NHANES, conducted by the Centers for Disease Control, is a nationally representative health survey of the non-institutionalized US population. Each year, the survey examines an estimated 5,000 individuals in counties across the country with both personal interviews conducted in the household and physical examinations performed in a mobile examination center. Interviews include demographic, socioeconomic, and health-related questions. This includes the Patient Health Questionnaire-9 (PHQ-9), a validated symptom instrument widely used for depression screening [18]. Female participants receive a reproductive health questionnaire as part of the interview [19].

### Study groups

We defined three study groups: control, antepartum, postpartum. Women who had a positive pregnancy test were included in the antepartum group. Women who reported a birth within the past twelve months, identified with the question, “How many months ago did you have your baby?”, and had a negative pregnancy test, were included in the postpartum group. The control group was comprised of women who had a negative pregnancy test and were not in the 12-month postpartum period.

### Outcome measurement

The primary outcome was depression prevalence, defined as PHQ-9 score ≥10 or self-reported current antidepressant use. Using a threshold of ≥ 10 is highly sensitive and specific for major depressive disorder, both in the general and perinatal populations [20–22]. Self-reported antidepressant use was defined as prescription medications taken in the past 30 days including bupropion, citalopram, desvenlafaxine, duloxetine, escitalopram, fluoxetine, imipramine, nortriptyline, paroxetine, sertraline, or venlafaxine. These antidepressants were chosen based on previous publications in perinatal depression [23,24]. Self-reported antidepressant use was included in the definition of prevalent depression because this may identify individuals living with depression whose symptoms are currently managed by their antidepressant treatment, as has been done in other studies assessing depression prevalence [25,26].

Additional outcomes included prevalence of: (i) moderate depressive symptoms or higher (PHQ-9 score ≥10), (ii) any depressive symptoms (PHQ-9 score ≥5) or antidepressant prescription, and (iii) total PHQ-9 score as a continuous outcome. Additional treatment outcomes evaluated included: (i) antidepressant prescription (with or without elevated PHQ-9 score) and (ii) mental health care service utilization, identified using the question, “During the past 12 months, have you seen or talked to a mental health professional such as a psychologist, psychiatrist, psychiatric nurse or clinical social worker about your health?” Throughout the manuscript, we refer to ‘moderate’ to ‘severe’ depressive symptoms, with or without antidepressant use, as depression.

A sensitivity analysis restricted to 2013–2019 leveraged the 2013 modification to NHANES where indication for each prescription was collected and coded according to International Classification of Diseases, Tenth Revision (ICD-10) codes. For this sensitivity outcome, depression was additionally defined as total PHQ-9 score of ≥ 10 or ICD-10 codes F32.9 (Major depressive disorder, single episode, unspecified) or F33.9 (Major depressive disorder, recurrent, unspecified) noted as prescription indications (regardless of antidepressant type) associated with a prescription.

### Covariate assessment

Information on age, race/ethnicity, education level, marital status, family income, health insurance status, and pregnancy history were collected during interviews. Per NHANES data derivation guidelines, Hispanic individuals were included in the “Hispanic” category of race/ethnicity regardless of race. We used race/ethnicity categories determined by NHANES, which included Hispanic, non-Hispanic White, non-Hispanic Black, and Other Race/Multiracial. Ratio of family income to poverty was derived by NHANES by dividing total annual family (or individual) income by poverty guidelines specific to the survey year, family size, and geographic location. We created a categorical variable based on federal assistance program eligibility criteria, with ≤1.3 indicating low income [27]. Body measurements were collected during the physical examination by trained health technicians. Body mass index (BMI) was calculated as weight in kilograms divided by height in meters squared.

### Statistical analysis

Descriptive statistics assessed participant characteristics and outcomes stratified by study group and aggregated across all data cycles. Weighted proportions and standard errors are reported for categorical variables, and means, standard deviations, medians, and interquartile ranges for continuous variables. We calculated 95% confidence intervals for all outcomes using the Korn-Graubald method for exact intervals [28,29].

Trends in outcomes by data cycle were assessed using logistic regression to identify linear trends over time. The prevalence of depression was assessed and stratified by study group and according to participant characteristics pooled across all NHANES years. We subsequently performed logistic regression adjusted for age, insurance type, race/ethnicity, education level, and marital status to estimate the association between prevalent depression and study group. As a sensitivity analysis to distinguish the effects of antidepressant use and PHQ-9 score, we performed logistic regression with PHQ-9 ≥ 10 only as the outcome. Since missing data were minimal, with only 32 participants missing values for one or more variables in our regression analysis, complete case analyses were performed.

As an exploratory analysis, item-level descriptive statistics were generated for individual PHQ-9 questions for control, antepartum, and postpartum women with the primary outcome for all cycles combined.

All analyses accounted for complex survey design, including oversampling, survey nonresponse, and post-stratification of NHANES using sample weights, strata, and primary sampling units included in the NHANES data. For analyses pooled across years, we followed NHANES Analytic Guidelines to construct weights for combined NHANES survey cycles [30]. All tests were two-sided and performed at the 0.05 significance level. The research protocol and statistical analysis plan were pre-registered on Open Science Framework (OSF.io) prior to data analysis [31]. All analyses were performed with R version 4.2.2 [32].

## Results

Across all data cycles, a total of 6181 participants met inclusion criteria, including: 5412 control group women, 314 antepartum women, and 455 postpartum women (STROBE diagram S1 Fig). Among the weighted study population participants, the median age was 32 [IQR = 25,38]. Fifty-nine percent of participants were White, 18.5% Hispanic, 13.4% Black, and 9.1% specified Other Race/Multiracial. The majority (78.3%) of participants reported having health insurance, with 58.8% reporting private insurance and 11.2% Medicaid. Twenty-seven percent had a family income 1.3 times the poverty level or lower (Table 1). Sixty-five percent of the control group reported ever being pregnant. The average time since delivery for postpartum women was 6.4 months (SD = 3.6).

**Table 1:** Descriptive characteristics by study group.

	Overall	Control	Antepartum	Postpartum
Unweighted Count	6181	5412	314	455
Age in years (Median [IQR])	32 [25,38]	32 [26,39]	28 [24,33]	28 [23,33]
Age in Years				
20-24	21.0 (1.0)	19.7 (1.1)	30.4 (3.1)	31.3 (2.6)
25-34	39.0 (0.9)	37.5 (0.9)	48 (3.5)	52.2 (2.9)
35-39	19.9 (0.6)	20.6 (0.7)	17.2 (2.7)	12.8 (2.2)
40-44	20.1 (0.8)	22.3 (0.9)	4.5 (2.0)	3.8 (1.0)
Race/Ethnicity				
Hispanic	18.5 (1.3)	18.0 (1.3)	22.2 (2.7)	22.4 (2.2)
Non-Hispanic White	59.0 (1.7)	59.7 (1.7)	49.1 (4.1)	56.2 (3.0)
Non-Hispanic Black	13.4 (0.9)	13.2 (0.9)	16.0 (2.0)	13.8 (1.7)
Other Race/Multiracial	9.1 (0.5)	9.0 (0.6)	12.7 (2.2)	7.5 (1.3)
Language				
English	92.9 (0.6)	93.0 (0.6)	91.8 (1.6)	92.1 (1.1)
Spanish	7.1 (0.6)	7.0 (0.6)	8.2 (1.6)	7.9 (1.1)
Education Level				
Less than 9th Grade	13.1 (0.6)	12.6 (0.7)	18.1 (2.2)	16.7 (2.0)
High School	18.7 (0.8)	18.3 (0.8)	17.4 (2.2)	24.5 (2.5)
Some College or AA Degree	36.4 (0.9)	36.8 (1.0)	34.0 (3.1)	33.0 (2.4)
College or above	31.7 (1.3)	32.3 (1.3)	30.6 (3.3)	25.8 (2.8)
Body Mass Index^1^ (kg/m^2^)				
<25	37.4 (1.0)	38.5 (1.1)	22.3 (2.4)	33.5 (3.0)
25- < 30	25.5 (0.7)	24.8 (0.7)	36.3 (4.0)	26.0 (2.3)
30- < 35	16.8 (0.5)	16.7 (0.6)	19.6 (2.7)	16.1 (2.1)
≥35	20.0 (0.7)	19.6 (0.8)	21.7 (2.7)	24.4 (2.3)
NA	0.3	0.4	0	0
Marital Status				
Married	46.4 (1.1)	44.3 (1.2)	64.5 (3.5)	60.3 (3.0)
Widowed	0.5 (0.1)	0.5 (0.1)	0 (0)	0.1 (0.1)
Divorced	7.2 (0.4)	7.8 (0.5)	2.2 (0.9)	2.7 (1.0)
Separated	2.9 (0.2)	3.0 (0.2)	2.2 (0.8)	1.6 (0.5)
Never Married	29.8 (1.1)	31.5 (1.1)	16.6 (2.2)	16.7 (2.0)
Living With Partner	13.3 (0.6)	12.9 (0.6)	14.4 (2.3)	18.6 (1.9)
NA	0	0	0.1	0
Ratio Family Income to Poverty Level				
≤1.3	26.9 (0.9)	26.1 (1.0)	25.7 (2.4)	37.4 (2.4)
>1.3-3.5	34.1 (0.9)	34.3 (1.0)	30.1 (3.4)	35.6 (2.7)
>3.5	32.9 (1.2)	33.6 (1.2)	35.8 (4.2)	21.5 (2.5)
NA	6.1	6	8.3	5.6
Health Insurance				
Yes	78.3 (0.8)	77.6 (0.9)	86.6 (2.1)	81.2 (2.0)
No	21.5 (0.8)	22.2 (0.9)	13.4 (2.1)	18.6 (2.0)
NA	0.1	0.2	0	0.1
Health Insurance Type				
Private	58.8 (1.1)	60.0 (1.1)	51.0 (3.8)	49 (3.2)
Medicaid	11.2 (0.7)	9.4 (0.6)	27.1 (3.0)	23.1 (3.0)
Other Insurance	8.0 (0.5)	7.9 (0.5)	8.4 (1.9)	8.9 (1.8)
Medicare	0.5 (0.1)	0.5 (0.1)	0 (0)	0.5 (0.3)
SCHIP	0 (0)	0 (0)	0.3 (0.3)	0 (0)
Military	1.5 (0.3)	1.5 (0.3)	0 (0)	2.1 (1.1)
State-Sponsored Health Plan	3.5 (0.4)	3.4 (0.4)	5.6 (1.4)	3.4 (1.0)
Other Government	2.1 (0.2)	2.1 (0.3)	1.7 (0.8)	2.7 (0.7)
Single Service Plan	0.3 (0.1)	0.3 (0.1)	0.8 (0.7)	0.2 (0.2)
No Insurance	21.5 (0.8)	22.2 (0.9)	13.4 (2.1)	18.6 (2.0)
NA	0.5	0.5	0.2	0.4

Values are weighted percentages (SE) or median [IQR]. Missing values are presented as NA (not available).

SE, standard error; IQR, interquartile range; AA, Associate in Arts; SCHIP, State Children’s Health Insurance Program.

1 Body mass index measured at physical examination.

Compared to the control group, antepartum and postpartum groups were younger, more likely to be married, have health insurance, and be on Medicaid. Postpartum women were less likely to be college educated and had lower family incomes.

### Outcomes among the entire study population

The prevalence of depression was 20.2% (95% CI 18.5–21.9), 9.7% (6.3–14.1), 12.8% (9.3–17.1) in the control, antepartum, and postpartum groups, respectively, pooled across all cycles. Moderate or severe depression defined by PHQ-9 ≥ 10 was 11.1% (9.9–12.4), 6.5% (4.0–9.7), and 6.4% (3.7–10.0) in the three groups, respectively. Overall, antidepressant use and mental health care service utilization were highest in the controls, followed by postpartum women, and lowest in antepartum women (Table 2). Depression prevalence varied by parity: among pregnant women, it was 11.0% in nulliparous and 6.4% in multiparous women. Among postpartum women, depression was 14.9% in first-time mothers and 11.7% in mothers with two or more live births.

**Table 2:** Outcomes stratified by study group.

	Control	Antepartum	Postpartum
Overall	5412	314	455
PHQ-9 ≥ 10 or Antidepressant	20.2 (18.5-21.9)	9.7 (6.3-14.1)	12.8 (9.3-17.1)
PHQ-9 ≥ 10	11.1 (9.9-12.4)	6.5 (4.0-9.7)	6.4 (3.7-10.0)
PHQ-9 ≥ 5 or Antidepressant	34.0 (32.2-35.8)	28.3 (21.4-36.0)	28.5 (23.7-33.7)
Antidepressant Use	12.2 (10.8-13.7)	4.6 (2.1-8.6)	7.4 (4.6-11.1)
Mental Health Services Past 12 Months	12.1 (10.9-13.5)	6.4 (3.5-10.4)	8.7 (5.8-12.6)
Total PHQ-9 Score			
Median [IQR]	2 [1,5]	3 [1,5]	2 [0, 5]
Mean (SD)	3.7 (4.4)	3.4 (3.6)	3.2 (3.8)
Count in 2013–2018^1^	2690	157	231
PHQ-9 ≥ 10 or Antidepressant	19.9 (17.6-22.3)	12.2 (6.8-19.8)	11.5 (6.5-18.3)
Antidepressant Use	11.8 (9.8-14.0)	7.0 (2.7-14.5)	8.0 (3.7-14.5)
PHQ-9 ≥ 10 or Antidepressant (Sensitivity^2^)	16.3 (14.2-18.6)	11.4 (6.2-18.6)	7.3 (3.7-12.6)
Antidepressant Use (Sensitivity^2^)	7.6 (6.1-9.4)	5.8 (1.9-13.1)	3.6 (1.1-8.6)

PHQ-9, Patient Health Questionnaire-9; IQR, interquartile range; SD, standard deviation.

All counts are unweighted. Values are weighted percentages with 95% confidence intervals, unless otherwise noted.

^1^The bottom panel shows outcomes for the data in 2013–2018 when medication indication (ICD-10 code) was provided.

^2^Sensitivity outcome is defined as PHQ-9 ≥ 10 or Antidepressant with ICD-10 code for depression.

### Treatment among women with prevalent depression

**Fig 1:**
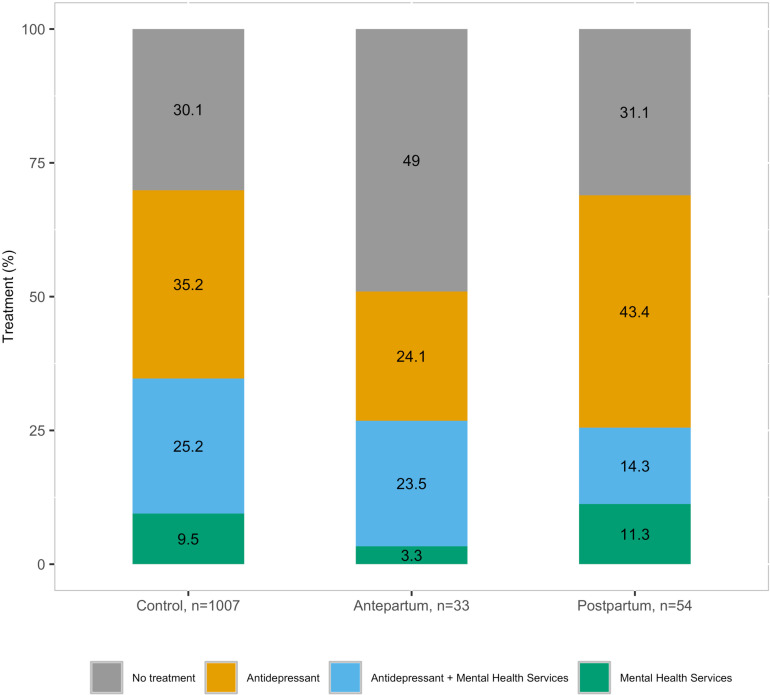
Treatment among women with prevalent depression (PHQ-9 ≥ 10 or antidepressant prescription).

Among those with prevalent depression, 69.9% control, 51% antepartum, and 68.9% postpartum women reported treatment with antidepressant use, mental health care services, or both. Antidepressant use alone was the most reported treatment among all groups (Fig 1). The differences in treatment rates were not statistically significant between control and antepartum women (p = 0.051), nor between control and postpartum women (p = 0.894). Mental health care service use was relatively low for individuals with PHQ-9 ≥ 10 without antidepressants (45.3% controls, 26.3% antepartum group, 37.4% postpartum group).

### Temporal trends in depression prevalence and treatment

**Fig 2:**
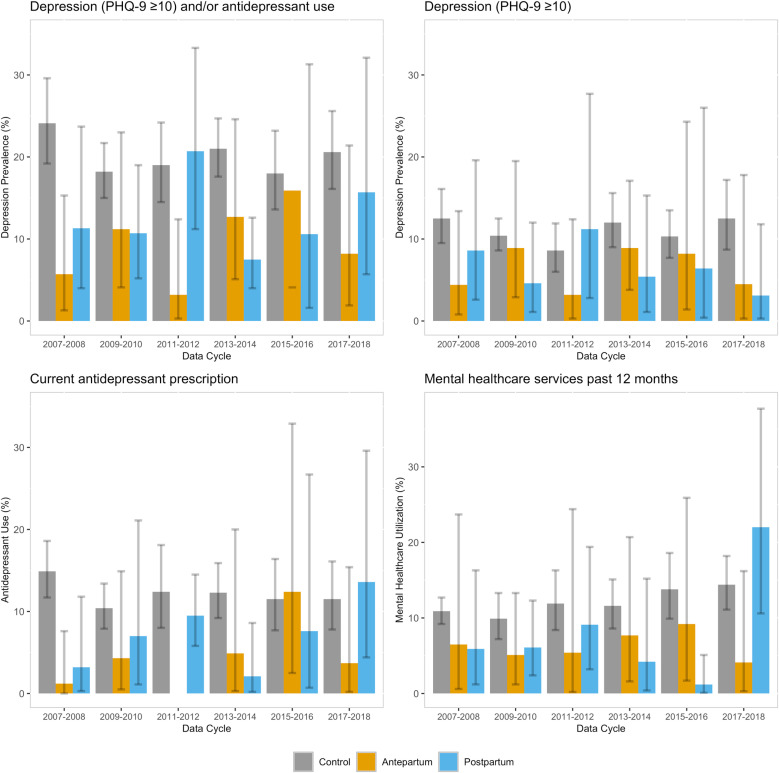
Temporal trends in primary and secondary depression outcomes, weighted percentages and 95% CIs.

Fig 2 shows trends in depression prevalence, antidepressant prescription, and mental health care utilization over a 12-year period. For the antepartum and postpartum groups, depression prevalence varied considerably across NHANES data cycles, as expected due to the relatively small sample sizes for both groups. For antepartum women, the highest prevalence of depression was in 2015–2016 (N = 58, 15.9%, 4.1–37.2), but we found no significant temporal trends. Antidepressant use increased significantly over time, peaking in 2015–2016 (N = 58, 12.4%, 2.5–32.9; linear trend p < 0.001).

In postpartum women, prevalence of depression was highest in 2011–2012 (N = 68, 20.7%, 11.2–33.3) and lowest in 2013–2014 (N = 76, 7.5%, 4.0–12.6), though these differences may reflect chance fluctuations given the small sample sizes. Use of antidepressants and mental health services similarly fluctuated, and we found no significant linear trend across the years, except for higher use of mental health care services in 2017–2018 (22.0% N = 76, 10.6–37.7, p = 0.021) (S1 Table).

In the control group, prevalence of depression and antidepressant use stayed constant over time. In contrast, mental health care service utilization increased linearly over time (p = 0.010), starting at 10.9% (9.2–12.7) in 2007–2008 and gradually increasing to 14.4% (11.1–18.2) in 2017–2018. All three outcomes were higher at almost every timepoint compared to the antepartum and postpartum groups. There were no statistically significant trends in total PHQ-9 score over time across all groups.

### Trends in depression prevalence by individual characteristics

Higher prevalence of depression was associated with increasing age, lower educational attainment, and White race. Those who were married or had private insurance had the lowest depression prevalence within their respective categorical variables. Hispanic ethnicity was associated with lower depression prevalence across the three groups, although the confidence intervals were imprecise for antepartum and postpartum women (Table 3).

**Table 3:** Depression prevalence (PHQ-9 ≥ 10 or antidepressant prescription) by study group characteristics.

	Control		Antepartum		Postpartum	
	Counts^1^	Weighted %, (95% CI)	Counts	Weighted %, (95% CI)	Counts	Weighted %, (95% CI)
Overall	5412	20.2 (18.5-21.9)	314	9.7 (6.3-14.1)	455	12.8 (9.3-17.1)
Age in Years						
20-24	1002	16.5 (13.5-19.9)	102	8.0 (3.2-15.9)	148	13.6 (7.1-22.7)
25-34	2015	16.7 (14.7-18.7)	153	9.0 (4.2-16.4)	230	13.4 (7.9-20.8)
35-39	1126	23.2 (20.4-26.3)	45	15.5 (4.3-35.6)	53	10.0 (0.5-39.0)
40-44	1269	26.5 (22.4-30.9)	14	6.4 (NA)	24	10.1 (NA^2^)
Race/Ethnicity						
Hispanic	1493	14.2 (12.3-16.3)	101	6.8 (2.5-14.3)	157	6.6 (3.0-12.3)
Non-Hispanic White	2032	23.6 (21.4-25.8)	89	10.3 (4.7-19.0)	159	16.7 (10.6-24.5)
Non-Hispanic Black	1151	17.1 (14.6-19.8)	75	11.7 (4.4-23.9)	91	10.4 (4.6-19.4)
Other Race/Multiracial	736	14.3 (10.5-18.7)	49	9.8 (1.5-29.2)	48	7.1 (0.8-24.0)
Language						
English	4776	20.9 (19.2-22.6)	275	10.2 (6.6-14.9)	393	13.1 (9.3-17.8)
Spanish	636	10.9 (8.3-14.0)	39	3.6 (0-31.7)	62	9.7 (2.2-24.8)
Education Level						
Less than 9th Grade	957	24.5 (21.0-28.3)	71	16.6 (7.4-30.1)	100	11.9 (5.0-2.7)
High School	1022	23.3 (19.5-27.5)	65	4.2 (0.6-13.7)	110	21.3 (12.2-33.0)
Some College or AA Degree	1989	21.3 (18.9-23.9)	108	11.3 (5.1-20.7)	146	14.6 (8.4-23.0)
College Degree or above	1443	15.3 (12.6-18.4)	70	7.0 (1.7-18.0)	99	3.2 (0.3-11.7)
Body Mass Index						
<25	1949	16.1 (14.2-18.2)	84	4.3 (1.1-10.9)	144	12.4 (5.9-21.9)
25- < 30	1342	19.7 (16.8-22.7)	98	10.2 (3.9-20.7)	130	8.1 (3.6-15.1)
30- < 35	967	24.2 (20.8-27.9)	65	13.2 (3.2-32.7)	79	11.4 (4.0-24.0)
≥35	1127	25.3 (21.9-29.0)	67	11.2 (4.5-22.0)	102	19.5 (8.9-34.8)
Marital Status						
Married	2264	16.9 (14.8-19.1)	170	7.1 (3.2-13.2)	246	8.4 (4.3-14.6)
Widowed	32	45.1 (4.6-92.0)	0	NA	1	0 (NA)
Divorced	410	34.7 (28.6-41.1)	10	33.2 (NA)	13	30.7 (NA)
Separated	217	39.9 (32.0-48.3)	9	28.9 (NA)	10	17.4 (NA)
Never Married	1795	18.2 (15.9-20.7)	67	8.9 (2.4-21.6)	87	22.9 (10.6-40.1)
Living With Partner	693	21.9 (18.2-26.0)	57	15.9 (7.1-29.0)	98	15.2 (6.9-27.5)
Ratio of Family Income to Poverty Level						
≤1.3	1843	26.0 (23.3-28.8)	109	15.7 (8.9-24.9)	209	12.2 (7.1-19.1)
>1.3-3.5	1818	19.3 (16.4-22.5)	99	11.8 (4.5-23.8)	145	8.0 (3.3-15.7)
>3.5	1351	17.2 (14.7-20.0)	73	2.9 (0.2-12.3)	74	16.6 (5.6-34.5)
Health Insurance						
Yes	3922	20.3 (18.5-22.1)	261	9.3 (5.6-14.3)	343	12.7 (8.5-18.1)
No	1483	19.8 (17.1-22.8)	53	12.4 (3.8-27.8)	111	13.3 (6.1-24.2)
Health Insurance Type						
Private	2770	17.6 (15.7-19.8)	131	7.5 (3.1-14.8)	181	8.3 (3.6-15.9)
Medicaid	670	32.4 (27.3-37.8)	101	9.6 (3.8-19.2)	117	14.3 (6.8-25.2)
Other Insurance	461	26.3 (21.2-31.9)	28	19.5 (NA)	44	33.6 (10.6-64.5)
No Insurance	1485	19.9 (17.1-22.8)	53	12.4 (3.8-27.8)	111	13.3 (6.1-24.2)

^1^Counts are unweighted. Weighted percentages are shown with 95% confidence intervals (CI).

^2^NA: Confidence intervals are suppressed for cells with counts less than 30.

After adjusting for these potential confounders in a multivariable regression, antepartum and postpartum women both had lower odds of prevalent depression compared to controls (Table 4; Antepartum: aOR 0.52, 0.33–0.81; Postpartum: aOR 0.67, 0.46–0.99). When the outcome was PHQ-9 ≥ 10 alone, these associations persisted (Antepartum: aOR 0.54, 0.32–0.89, Postpartum: aOR 0.50, 0.28–0.90).

**Table 4:** Adjusted logistic regression results for primary outcome of depression prevalence.

	aOR	Lower 95% CI	Upper 95% CI	p
Study Group				
Control	Reference			
Antepartum	0.52	0.33	0.81	0.005
Postpartum	0.67	0.46	0.99	0.042
Age in Years				
20-24	Reference			
25-34	1.17	0.91	1.51	0.224
35-39	1.78	1.36	2.34	<0.001
40-44	2.12	1.59	2.83	<0.001
Health Insurance				
Private	Reference			
Medicaid	1.89	1.44	2.48	<0.001
Other Insurance	1.67	1.26	2.21	<0.001
No Insurance	1.18	0.96	1.45	0.114
Race/Ethnicity				
Hispanic	Reference			
Non-Hispanic White	2.46	1.99	3.03	<0.001
Non-Hispanic Black	1.21	0.95	1.54	0.119
Other Race/Multiracial	1.38	0.96	1.97	0.081
Education Level				
Less than High School	Reference			
High School	0.95	0.74	1.22	0.712
Some College	0.86	0.67	1.11	0.240
College or above	0.58	0.44	0.77	<0.001
Marital Status				
Married	Reference			
Widowed	2.92	1.09	7.8	0.033
Divorced	2.24	1.64	3.06	<0.001
Separated	3.15	2.21	4.49	<0.001
Never Married	1.48	1.2	1.84	0.001
Living With Partner	1.56	1.21	2.02	0.001

Unweighted sample size for regression is 6,149 participants (32 excluded missing values for one or more variables).

aOR, adjusted odds ratio; CI, confidence interval.

### Sensitivity analysis

When antidepressant use was defined using ICD-10 codes for depression available in 2013–2018, lower depression prevalence was observed across all groups. During this period, depression prevalence using the primary outcome was 19.9% (17.6–22.3), 12.2% (6.8–19.8), and 11.5% (6.5–18.3) in the control, antepartum, and postpartum groups, respectively. In the sensitivity analysis, depression prevalence decreased to 16.3% (14.2–18.6), 11.4% (6.2–18.6), and 7.3% (3.7–12.6) in the three groups, respectively (Table 2, bottom panel). Out of the 274 participants with antidepressant prescriptions during this period, 63.5% had an accompanying depression ICD-10 code. In most cases where depression was not coded as the reason for prescription, anxiety disorder was cited.

The relationships between prevalent depression and educational attainment, income, race/ethnicity, and marital status remained the same. Depression prevalence no longer differed substantially by age (S2 Table). In the adjusted regression, postpartum women had lower odds of prevalent depression compared to the control (aOR 0.41, 0.20–0.84), while antepartum women had imprecise odds (aOR 0.71, 0.37–1.36) (S3 Table).

### PHQ-9 itemized responses

In the exploratory analysis of item-level PHQ-9 responses from women with prevalent depression (n = 1007 control, n = 33 antepartum, and n = 54 postpartum), the two most common symptoms of depression across study groups were feeling tired or having little energy (85.2%, 81.8%, and 87.0% in control, antepartum, and postpartum women, respectively) and trouble sleeping or sleeping too much (74.3%, 75.6%, 75.3%) (Table 5).

**Table 5:** PHQ-9 individual item responses by group for women with prevalent depression.

	Control	Antepartum	Postpartum	ASD^1^
Unweighted Count w/ Primary Outcome	1007	33	54	
Have little interest in doing things				0.407
Not at all	37.2 (2.0)	33.5 (9.9)	32.8 (8.5)	
Several days	35.9 (1.8)	30.9 (9.6)	44.7 (9.3)	
More than half the days	15.6 (1.2)	7.8 (6.1)	14.3 (6.0)	
Nearly every day	11.3 (1.3)	27.7 (9.1)	8.2 (4.1)	
Feeling down, depressed, or hopeless				0.300
Not at all	33.6 (2.2)	20.7 (8.7)	34.1 (8.7)	
Several days	36.3 (1.7)	36.4 (9.4)	38.4 (8.4)	
More than half the days	14.5 (1.4)	13.3 (3.6)	10.5 (3.6)	
Nearly every day	15.6 (1.4)	29.5 (8.4)	17.0 (6.5)	
Trouble sleeping or sleeping too much				0.236
Not at all	25.7 (1.7)	24.4 (10.0)	24.6 (6.3)	
Several days	26.2 (1.4)	26.7 (8.2)	21.2 (6.0)	
More than half the days	18.4 (1.3)	11.1 (4.5)	22.6 (7.1)	
Nearly every day	29.7 (2.0)	37.8 (9.2)	31.5 (8.2)	
Feeling tired or having little energy				0.267
Not at all	14.9 (1.4)	18.2 (9.7)	11.0 (6.1)	
Several days	32.1 (1.6)	25.5 (7.9)	34.0 (9.3)	
More than half the days	21.1 (1.4)	12.2 (5.6)	19.2 (6.4)	
Nearly every day	32.0 (1.6)	44.1 (9.4)	35.8 (8.2)	
Poor appetite or overeating				0.379
Not at all	37.2 (2.1)	35.6 (10.5)	35.5 (7.8)	
Several days	25.6 (1.9)	18.3 (6.8)	37.0 (7.1)	
More than half the days	17.6 (1.5)	14.4 (8.3)	14.8 (6.0)	
Nearly every day	19.6 (1.3)	31.7 (8.9)	12.7 (5.7)	
Feeling bad about yourself				0.457
Not at all	42.5 (2.0)	40.6 (10.4)	38.4 (8.6)	
Several days	28.8 (1.8)	38.7 (9.7)	39.1 (8.1)	
More than half the days	14.5 (1.2)	1.9 (1.9)	15.3 (5.8)	
Nearly every day	14.1 (1.3)	18.8 (7.8)	7.2 (5.3)	
Trouble concentrating on things				0.323
Not at all	46.4 (2.3)	51.2 (9.5)	39.0 (7.9)	
Several days	27.0 (1.9)	29.0 (7.6)	29.9 (7.5)	
More than half the days	13.7 (1.2)	4.4 (3.2)	7.3 (3.8)	
Nearly every day	13.0 (1.1)	15.3 (6.0)	23.9 (7.5)	
Moving or speaking slowly or too fast				0.389
Not at all	64.4 (1.6)	74.7 (7.5)	60.7 (8.4)	
Several days	19.5 (1.3)	22.2 (7.1)	26.5 (7.6)	
More than half the days	10.3 (1.1)	0 (0)	5.9 (3.4)	
Nearly every day	5.7 (0.7)	3.1 (2.2)	6.8 (3.9)	
Thoughts you would be better off dead				0.228
Not at all	85.9 (1.4)	91.0 (3.9)	89.7 (4.4)	
Several days	9.8 (1.2)	5.1 (2.9)	9.0 (4.2)	
More than half the days	2.5 (0.6)	2.1 (2.1)	0 (0)	
Nearly every day	1.7 (0.4)	1.9 (1.9)	1.2 (1.2)	

Values are weighted percentages (standard error).

^1^Absolute standardized difference (ASD). Values of 0.2, 0.5, and 0.8 correspond to small, moderate, and large differences between groups.

“Thoughts you would be better off dead” was present in some degree (answers ranging from several days to nearly every day the past two weeks) in 3.7%, 1.7%, and 2.9% of the overall population of control, antepartum, and postpartum women, respectively. In contrast, suicidal ideation was much higher in those with prevalent depression: 14.1%, 9.0%, 10.3%, in the three groups, respectively.

## Discussion

In a nationally representative sample of reproductive age women in 2007–2018, we found that the prevalence of depression, defined as PHQ-9 ≥ 10 or current antidepressant prescription, was 20.2% in controls (95% CI 18.5–21.9), 9.7% in antepartum women (6.3–14.1), and 12.8% in postpartum women screened within 12 months of childbirth (9.3–17.1). These differences persisted in adjusted analyses and sensitivity analyses. For those meeting criteria for prevalent depression, control and postpartum groups showed similar rates of antidepressant or mental health care service utilization (70%), while antepartum women had the lowest treatment rate (51%).

No significant temporal trends in depression prevalence were observed across assessment waves, though we lacked statistical power to detect trends in antepartum and postpartum women. Mental health care service utilization increased substantially for postpartum women in the last data cycle (2017–2018). Across both primary and sensitivity analyses, married status was protective against depression for control and postpartum women.

### Perinatal women have lower depression prevalence and lower rates of treatment

We found that antepartum and postpartum women had lower rates of depression compared to the control group, even after adjusting for confounding factors. This finding persisted even when examining PHQ-9 ≥ 10 alone, indicating that higher antidepressant use in the control group does not completely explain the findings. Two previous studies that used nationally representative data from 2005–2009 also found higher rates of depression in non-pregnant women compared to pregnant women, but the difference was not statistically significant [33,34]. However, both studies did not distinguish postpartum from non-pregnant, which could have artificially decreased depression prevalence in non-pregnant women. Similar to our study, a cross-sectional study in China found that pregnant women had 77% lower odds of PHQ-9 ≥ 10 than non-pregnant women, although this was specifically during the COVID-19 pandemic [35]. Our findings indicate lower prevalence of depressive symptoms among pregnant and postpartum women compared to non-pregnant controls. The PHQ-9 may not be adequate at screening for depression in perinatal populations, potentially underestimating the depression burden in antepartum and postpartum women [36]. Cohort studies with longitudinal follow-up are needed to explain these findings. Finally, note that most women with postpartum depression experience onset in the early postpartum period, which could lead to an underestimate of PPD prevalence in our sample, given that the average postpartum time in our sample was 6 months [37,38]. However, our prevalence estimates line up with past studies, suggesting instead that PPD identified in our study is unresolved.

Pregnant women with depression used antidepressant or mental health care services less compared with non-pregnant women in our study. Ko et al. found that 50% of pregnant women with prevalent depression reported mental health treatment (defined as prescription medication, counseling, or inpatient care), compared to 54% of non-pregnant women in 2005–2009 [33]. Our more recent data suggest that this treatment gap has widened substantially over time, with treatment rates in pregnant women still at only 51% compared to 70% for non-pregnant women, although we were not powered to see a statistically significant effect (p = 0.051). While most postpartum women are screened for depression at least once after birth, antepartum women do not generally receive screening as part of prenatal care, which could be a factor in treatment rates. Concern around the potential negative effects of maternal antidepressant use on the growing fetus likely also plays a role in lower pharmacological treatment rates [39–41].

### Mental health care services increased substantially for postpartum women

We found that mental health care services utilization doubled from less than 10% in previous data cycles to 22% in 2017–2018 for postpartum women. In January 2016, the US Preventive Services Task Force (USPSTF) recommended universal depression screening for pregnant and postpartum women [42]. Additionally, the USPSTF, noting potential harms to the fetus and breastfeeding newborn from antidepressants, encouraged clinicians to consider evidence-based psychosocial treatments, such as cognitive behavioral therapy, as a first-line treatment for perinatal depression. This may have contributed to the increase in mental health services we observed in the ensuing years, but most perinatal women with prevalent depression continued to report antidepressant use without additional mental health care.

Psychological treatments remain far from universally accessible. We found that across all years, utilization of mental health care services in the past 12 months among those with prevalent depression was 34.7%, 26.8%, and 25.6% for control, antepartum, and postpartum women, respectively. In response to the USPSTF guidelines, one article outlines the need for infrastructure changes in health care settings to meet mental health care demands for perinatal women, similar to how obstetric clinics screen for and treat gestational diabetes mellitus [43].

### Married women have lower depression prevalence

We found that married women had lower depression prevalence than unmarried women in postpartum and control groups, even after accounting for confounding variables. For many mothers, an intimate partner is the primary source of social support after childbirth. Low levels of social support are one of the predominant risk factors for postpartum depression, and are even more impactful than pregnancy- and delivery-related complications and socioeconomic status [44–49].

Consistent with our findings, previous research has found that mothers in stable marriages have superior physical and mental health compared to unmarried mothers within a year of childbirth, which was not explained by greater socioeconomic resources [49]. A meta-analysis found that lower marital satisfaction and being unmarried are linked to higher rates of postpartum depression [45]. This aligns with the broader narrative around the positive effects of marriage on health outcomes beyond parenthood, including better mental health over the life course [50,51]. Future studies should consider marital status as a proxy for social support, especially in datasets where more precise social support measurements are difficult or impossible to obtain, such as administrative claims data and electronic health records.

### Suicidal ideation

We found that suicidal ideation was present in about 10% of perinatal women with prevalent depression, compared to 2–3% of perinatal women in the overall population. We also found high rates of sleep disturbance in our sample, which is an evidence-based risk factor for suicidal behaviors across the lifespan, and associated with postpartum depression [52–54]. Adequate sleep and good self-reported sleep quality are important factors in assessing quality of postpartum recovery [55], and may be a novel opportunity for prevention [52,56]. Given that mental health conditions are now the leading cause of pregnancy-related mortality in the US, future research is critical to enhance novel screening, treatment, and referral strategies that may guide depression and suicide prevention efforts in perinatal women specifically [57].

### Limitations and strengths

Limitations included reduced power to analyze trends across time due to small sample sizes of postpartum and pregnant women with prevalent depression per data cycle, and a cross-sectional study design. Future prospective study of epidemiological trends in depression prevalence are recommended to establish temporality between perinatal status and depression onset. Next, our primary analysis defined antidepressant use broadly, allowing for some amount of misclassification given antidepressant medications have other indications, especially in non-perinatal populations. However, in a sensitivity analysis of years that included diagnosis codes for antidepressant prescriptions, our conclusions were similar compared to defining antidepressant use broadly. As an additional limitation, mental health care services were defined dichotomously (yes/no) and over a 12-month period, preventing study of treatment linked to pregnancy or postpartum directly. Given the well-established link between socioeconomic status and depression, we initially planned to adjust for the income-to-poverty ratio from NHANES data. However, because this variable had a relatively high rate of missingness, we used insurance type as a proxy instead. Finally, several measurements were limited to self-report, and key confounding factors, such as a history of mental health conditions and adverse pregnancy and delivery outcomes that increase depression risk, were not collected.

Despite these limitations, our study’s greatest strength is that the participants comprise a socioeconomically, racially, and ethnically diverse group of individuals that are representative of reproductive-age women in the United States. This is the first study to evaluate antepartum and postpartum depression trends in a cohort of women with private and public insurance, increasing the generalizability of our findings. NHANES is known for its comprehensive data collection and rigorous standards, which allow for the analysis of various health indicators with limited information bias. All analyses were pre-registered to limit false positives in our findings.

## Conclusion

In conclusion, over a twelve-year period in the US, the prevalence of antepartum depression was 10% and postpartum depression was 13%, both of which were significantly lower compared to depression in reproductive-age women who were neither pregnant nor postpartum (20%). Rates of recent mental health care services doubled in postpartum women over time, perhaps reflecting the impact of USPSTF guidelines on treatment for depression. Married women had significantly lower rates of prevalent depression: marital status may be acting as a proxy for social support, one of the most important predictors of postpartum depression.

Future research should evaluate the causal effect of USPSTF and American College of Obstetrics and Gynecology depression screening guidelines on depression and treatment trends in perinatal populations within the US, particularly whether such guidelines led to meaningful change in mental health services access and uptake and ultimately decreased depressive symptoms for antepartum and postpartum individuals with depression.

## Supporting Information

S1 Fig STROBE diagram. (TIF)

S1 Table Trends in depression and treatment outcomes stratified by study group over time. (DOCX)

S2 Table Depression prevalence with sensitivity outcome (PHQ-9 ≥ 10 or antidepressant prescription with ICD-10 code) by study group characteristics. (DOCX)

S3 Table Adjusted logistic regression results for sensitivity depression prevalence outcome. (DOCX)
