# Supplementary figures and images for: Trends in prevalence and treatment of antepartum and postpartum depression in the United States: Data from the national health and nutrition examination survey (NHANES) 2007 to 2018
Source: PLoS One. 2025 Apr 30;20(4):e0322536. doi: 10.1371/journal.pone.0322536 (PMC12043162; doi:10.1371/journal.pone.0322536)

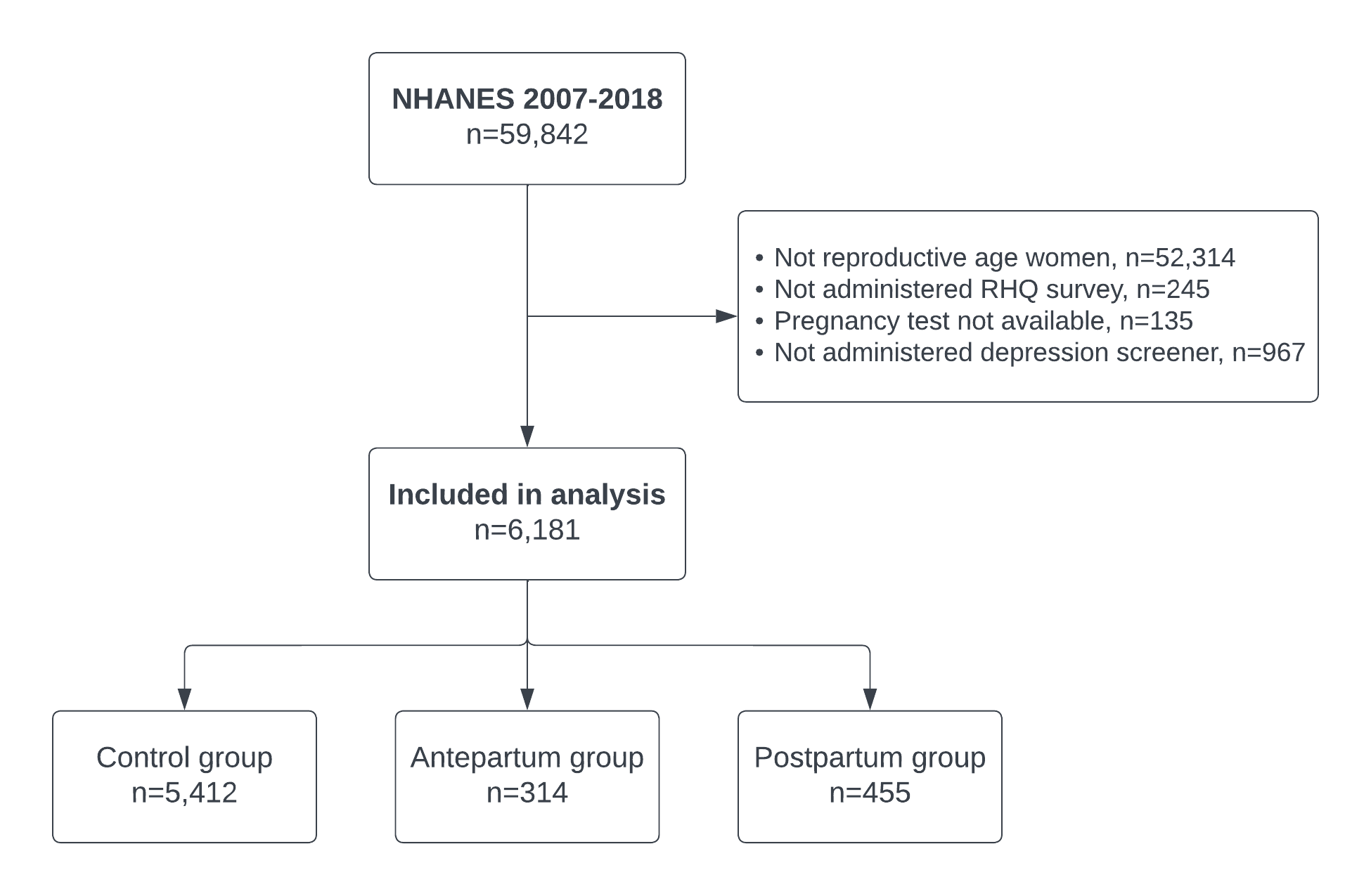

Supplement: S1 Fig — (TIF) [file pone.0322536.s001.tif]
