# Supplementary material for: Trends in prevalence and treatment of antepartum and postpartum depression in the United States: Data from the national health and nutrition examination survey (NHANES) 2007 to 2018
Source: PLoS One. 2025 Apr 30;20(4):e0322536. doi: 10.1371/journal.pone.0322536 (PMC12043162; doi:10.1371/journal.pone.0322536)
Supplement: S1 Table — (DOCX) [file pone.0322536.s002.docx]

**Supplemental Table 1. Trends in depression and treatment outcomes stratified by study group over time.**

| POSTPARTUM | 2007-2008 | 2009-2010 | 2011-2012 | 2013-2014 | 2015-2016 | 2017-2018 | P Trend^a^ |
| --- | --- | --- | --- | --- | --- | --- | --- |
| Unweighted Count | 78 | 78 | 68 | 76 | 79 | 76 |  |
| Depression (PHQ-9 ≥10) or Antidepressant Use | 11.3 (4.0-23.7) | 10.7 (5.2-19.0) | 20.7 (11.2-33.3) | 7.5 (4.0-12.6) | 10.6 (1.6-31.3) | 15.7 (5.7-32.1) | 0.848 |
| Depression (PHQ-9 ≥10) | 8.6 (2.6-19.6) | 4.6 (1.1-12.0) | 11.2 (2.8-27.7) | 5.4 (1.1-15.3) | 6.4 (0.4-26.0) | 3.1 (0.3-11.8) | 0.328 |
| Total PHQ-9 Score |  |  |  |  |  |  |  |
| Median [IQR] | 2 [0, 5] | 2 [1, 4] | 2 [0, 5] | 2 [0, 3] | 2 [1, 5] | 2 [0, 5] |  |
| Mean (SD) | 3.4 (3.9) | 3.0 (3.3) | 4.1 (5.6) | 2.7 (3.7) | 2.9 (3.1) | 3.0 (3.0) | 0.116 |
| Any Depressive Symptoms (PHQ-9 ≥5) or Antidepressant Use | 30.3 (16.7-47.0) | 25.8 (17.6-35.4) | 31.4 (21.5-42.8) | 18.2 (8.5-32.0) | 31.1 (17.0-48.3) | 34.2 (20.2-50.4) | |
| Mental Health Services Past 12 Months | 5.9 (1.2-16.3) | 6.1 (2.4-12.3) | 9.1 (3.2-19.4) | 4.2 (0.4-15.2) | 1.2 (0.1-5.1) | 22.0 (10.6-37.7) | 0.662 |
| Antidepressant Use | 3.2 (0.3-11.8) | 7.0 (1.1-21.1) | 9.5 (5.8-14.5) | 2.1 (0.2-8.6) | 7.6 (0.7-26.7) | 13.6 (4.4-29.6) | 0.203 |
| ANTEPARTUM | **2007-2008** | **2009-2010** | **2011-2012** | **2013-2014** | **2015-2016** | **2017-2018** | **P Trend^a^** |
| Unweighted Count | 53 | 59 | 45 | 54 | 58 | 45 |  |
| Depression (PHQ-9 ≥10) or Antidepressant Use | 5.7 (1.3-15.3) | 11.2 (4.1-23.0) | 3.2 (0.3-12.4) | 12.7 (5.1-24.6) | 15.9 (4.1-37.2) | 8.2 (1.9-21.4) | 0.284 |
| Depression (PHQ-9 ≥10) | 4.4 (0.8-13.4) | 8.9 (2.9-19.5) | 3.2 (0.3-12.4) | 8.9 (3.8-17.1) | 8.2 (1.4-24.3) | 4.5 (0.3-17.8) | 0.868 |
| Total PHQ-9 Score |  |  |  |  |  |  |  |
| Median [IQR] | 3 [1, 4] | 4 [1, 6] | 1 [0, 3] | 3 [1, 4] | 2 [1, 4] | 3 [1, 5] |  |
| Mean (SD) | 3.6 (3.3) | 4.0 (3.8) | 2.3 (2.8) | 3.8 (3.8) | 3.2 (3.6) | 3.6 (3.7) | 0.575 |
| Any Depressive Symptoms (PHQ-9 ≥5) or Antidepressant Use | 22.5 (9.6-41.0) | 42.5 (22.6-64.3) | 13.7 (3.9-31.5) | 27.5 (12.8-46.9) | 30.9 (13.6-53.2) | 30.5 (10.8-57.3) | |
| Mental Health Services Past 12 Months | 6.5 (0.6-23.7) | 5.1 (1.2-13.3) | 5.4 (0.2-24.4) | 7.7 (1.6-20.7) | 9.2 (1.7-25.9) | 4.1 (0.3-16.2) | 0.981 |
| Antidepressant Use | 1.2 (0-7.6) | 4.3 (0.5-14.9) | 0 (0) | 4.9 (0.3-20.0) | 12.4 (2.5-32.9) | 3.7 (0.2-15.4) | <0.001 |
| CONTROL | **2007-2008** | **2009-2010** | **2011-2012** | **2013-2014** | **2015-2016** | **2017-2018** | **P Trend^a^** |
| Unweighted Count | 903 | 1002 | 817 | 960 | 901 | 829 |  |
| Depression (PHQ-9 ≥10) or Antidepressant Use | 24.1 (19.2-29.6) | 18.2 (15.0-21.7) | 19.0 (14.5-24.2) | 21.0 (17.6-24.7) | 18.0 (13.6-23.2) | 20.6 (16.1-25.6) | 0.396 |
| Depression (PHQ-9 ≥10) | 12.5 (9.5-16.1) | 10.4 (8.6-12.5) | 8.6 (6.0-11.9) | 12.0 (9.0-15.6) | 10.3 (7.7-13.5) | 12.5 (8.7-17.2) | 0.226 |
| Total PHQ-9 Score |  |  |  |  |  |  |  |
| Median [IQR] | 2 [0, 5] | 2 [1, 6] | 2 [0, 4] | 2 [0, 5] | 2 [1, 5] | 2 [1, 5] |  |
| Mean (SD) | 3.9 (4.7) | 3.8 (4.4) | 3.3 (4.2) | 3.7 (4.6) | 3.7 (4.4) | 3.7 (4.4) | 0.912 |
| Any Depressive Symptoms (PHQ-9 ≥5) or Antidepressant Use | 36.4 (30.9-40.2) | 35.9 (31.7-40.3) | 31.5 (26.3-37.0) | 33.7 (30.5-37.1) | 34.1 (29.4-39.0) | 32.2 (27.4-37.3) | |
| Mental Health Services Past 12 Months | 10.9 (9.2-12.7) | 9.9 (7.2-13.3) | 11.9 (8.4-16.3) | 11.6 (8.6-15.1) | 13.8 (9.9-18.6) | 14.4 (11.1-18.2) | 0.012 |
| Antidepressant Use | 14.9 (11.7-18.6) | 10.4 (7.9-13.4) | 12.4 (8.0-18.1) | 12.3 (9.2-15.9) | 11.5 (7.7-16.4) | 11.5 (7.8-16.1) | 0.377 |
