# Supplementary material for: Trends in prevalence and treatment of antepartum and postpartum depression in the United States: Data from the national health and nutrition examination survey (NHANES) 2007 to 2018
Source: PLoS One. 2025 Apr 30;20(4):e0322536. doi: 10.1371/journal.pone.0322536 (PMC12043162; doi:10.1371/journal.pone.0322536)
Supplement: S2 Table — (DOCX) [file pone.0322536.s003.docx]

**Supplemental Table 2. Depression prevalence with sensitivity outcome (PHQ-9 ≥10 or antidepressant prescription with ICD-10 code) by study group characteristics.**

|  | Control |  | Antepartum | | Postpartum | |
| --- | --- | --- | --- | --- | --- | --- |
|  | Counts^1^ | Weighted %, (95% CI) | Counts | Weighted %,  (95% CI) | Counts | Weighted %, (95% CI) |
| Overall | 2690 | 19.9 (17.6-22.3) | 157 | 12.2 (6.8-19.8) | 231 | 11.5 (6.5-18.3) |
| Age in Years |  |  |  |  |  |  |
| 20-24 | 473 | 18.6 (14.0-24.0) | 51 | 3.6 (0.1-17.3) | 62 | 11.8 (3.2-27.8) |
| 25-34 | 1010 | 13.8 (11.3-16.8) | 77 | 14.5 (5.4-29.4) | 126 | 6.8 (2.4-14.5) |
| 35-39 | 562 | 17.1 (13.9-20.8) | 20 | 21.9 (NA) | 28 | 0 (NA) |
| 40-44 | 645 | 17.8 (13.7-22.4) | 9 | 8.9 (NA) | 15 | 4.0 (NA) |
| Race/Ethnicity |  |  |  |  |  |  |
| Hispanic | 729 | 11.0 (8.5-13.9) | 42 | 6.7 (0.5-25.0) | 70 | 2.2 (0.2-8.7) |
| Non-Hispanic White | 895 | 18.8 (15.8-22.0) | 46 | 15.5 (5.8-31.1) | 82 | 9.0 (3.3-18.8) |
| Non-Hispanic Black | 582 | 16.0 (12.5-20.1) | 39 | 10.7 (2.0-29.6) | 44 | 6.3 (0.7-21.4) |
| Other Race/Multiracial | 484 | 13.5 (9.1-19.0) | 30 | 3.0 (0-23.1) | 35 | 8.6 (0.6-32.7) |
| Language |  |  |  |  |  |  |
| English | 2404 | 16.9 (14.7-19.3) | 146 | 11.6 (6.4-19.0) | 203 | 7.3 (3.5-13.2) |
| Spanish | 286 | 8.1 (4.9-12.4) | 11 | 6.3 (NA) | 28 | 6.3 (NA) |
| Education Level |  |  |  |  |  |  |
| Less than 9th Grade | 409 | 20.8 (15.6-26.8) | 34 | 14.2 (2.2-40.1) | 42 | 10.5 (1.8-30.2) |
| High School | 500 | 20.3 (14.8-26.7) | 33 | 4.0 (0-24.7) | 52 | 10.9 (2.3-28.8) |
| Some College or AA Degree | 1020 | 18.6 (15.2-22.3) | 54 | 16.0 (5.4-33.5) | 81 | 9.2 (2.5-22.0) |
| College or above | 761 | 10.2 (7.2-13.9) | 36 | 8.8 (1.0-28.7) | 56 | 0 (NA) |
| Body Mass Index |  |  |  |  |  |  |
| <25 | 930 | 12.5 (10.0-15.3) | 41 | 5.6 (0.6-19.4) | 72 | 8.3 (2.1-20.6) |
| 25-<30 | 641 | 16.1 (12.3-20.4) | 45 | 11.8 (1.1-39.4) | 64 | 8.2 (2.1-20.4) |
| 30-<35 | 491 | 19.4 (14.2-25.5) | 36 | 16.2 (0.3-65.7) | 41 | 0 (NA) |
| ≥35 | 612 | 20.8 (16.6-25.4) | 35 | 10.9 (2.0-30.0) | 54 | 9.3 (1.3-28.6) |
| Marital Status |  |  |  |  |  |  |
| Married | 1114 | 10.0 (7.5-13.0) | 84 | 10.0 (3.3-22.0) | 128 | 1.5 (0.4-4.1) |
| Widowed | 19 | 54.2 (NA) | 0 | NA | 0 | NA |
| Divorced | 183 | 30.0 (21.1-40.1) | 7 | 42.5 (NA) | 8 | 13.1 (NA) |
| Separated | 104 | 38.2 (26.0-51.7) | 6 | 10.5 (NA) | 8 | 20.1 (NA) |
| Never Married | 895 | 16.7 (13.4-20.5) | 29 | 6.0 (NA) | 42 | 26.2 (7.7-54.1) |
| Living with Partner | 375 | 20.8 (15.8-26.6) | 31 | 14.9 (1.7-45.3) | 45 | 4.8 (0.6-16.4) |
| Ratio of Family Income to Poverty Level |  |  |  |  |  |  |
| ≤1.3 | 884 | 23.4 (19.5-27.7) | 60 | 15.9 (7.2-28.8) | 98 | 6.4 (2.2-13.9) |
| >1.3-3.5 | 917 | 15.8 (12.8-19.2) | 50 | 12.5 (1.6-38.2) | 81 | 0.9 (0.1-3.4) |
| >3.5 | 675 | 11.3 (8.4-14.9) | 31 | 4.4 (0-33.0) | 39 | 14.5 (2.3-40.5) |
| Health Insurance |  |  |  |  |  |  |
| Yes | 2051 | 15.6 (13.3-18.1) | 135 | 10.5 (5.0-18.9) | 182 | 5.7 (2.3-11.5) |
| No | 635 | 19.0 (15.1-23.6) | 22 | 17.6 (NA) | 48 | 15.0 (3.3-37.6) |
| Health Insurance Type |  |  |  |  |  |  |
| Private | 1387 | 12.2 (9.9-14.9) | 64 | 9.5 (2.2-24.6) | 102 | 4.1 (0.5-14.4) |
| Medicaid | 402 | 27.0 (21.0-33.8) | 56 | 5.9 (0.9-18.1) | 61 | 7.7 (1.7-20.5) |
| Other insurance | 243 | 23.1 (16.1-31.3) | 15 | 33.6 (NA) | 18 | 11.2 (NA) |
| No insurance | 637 | 19.2 (15.2-23.7) | 22 | 17.6 (NA) | 48 | 15.0 (3.3-37.6) |

^1^ Counts are unweighted. Weighted percentages are shown with 95% confidence intervals (CI). Confidence intervals are suppressed for cells with counts less than 30.
