# Supplementary material for: Trends in prevalence and treatment of antepartum and postpartum depression in the United States: Data from the national health and nutrition examination survey (NHANES) 2007 to 2018
Source: PLoS One. 2025 Apr 30;20(4):e0322536. doi: 10.1371/journal.pone.0322536 (PMC12043162; doi:10.1371/journal.pone.0322536)
Supplement: S3 Table — (DOCX) [file pone.0322536.s004.docx]

**Supplemental Table 3. Adjusted logistic regression results for sensitivity depression prevalence outcome.**

|  | **aOR** | **Lower 95% CI** | **Upper 95% CI** | **p** |
| --- | --- | --- | --- | --- |
| **Study Group** |  |  |  |  |
| Control | Reference |  |  |  |
| Antepartum | 0.71 | 0.37 | 1.36 | 0.292 |
| Postpartum | 0.41 | 0.2 | 0.84 | 0.016 |
| **Age in years** |  |  |  |  |
| 20-24 | Reference |  |  |  |
| 25-34 | 0.92 | 0.62 | 1.36 | 0.678 |
| 35-39 | 1.16 | 0.76 | 1.75 | 0.482 |
| 40-44 | 1.19 | 0.76 | 1.86 | 0.435 |
| **Health Insurance** |  |  |  |  |
| Private | Reference |  |  |  |
| Medicaid | 1.8 | 1.15 | 2.81 | 0.012 |
| Other insurance | 1.91 | 1.33 | 2.73 | 0.001 |
| No insurance | 1.66 | 1.16 | 2.38 | 0.007 |
| **Race/Ethnicity** |  |  |  |  |
| Hispanic | Reference |  |  |  |
| Non-Hispanic White | 2.74 | 1.93 | 3.89 | <0.001 |
| Non-Hispanic Black | 1.47 | 1.01 | 2.14 | 0.043 |
| Other Race/Multiracial | 1.91 | 1.15 | 3.18 | 0.014 |
| **Education Level** |  |  |  |  |
| Less than high school | Reference |  |  |  |
| High school | 0.94 | 0.61 | 1.46 | 0.778 |
| Some college or AA | 0.91 | 0.6 | 1.37 | 0.628 |
| College or above | 0.5 | 0.32 | 0.8 | 0.005 |
| **Marital Status** |  |  |  |  |
| Married | Reference |  |  |  |
| Widowed | 8.46 | 2.41 | 29.67 | 0.002 |
| Divorced | 3.31 | 1.93 | 5.67 | <0.001 |
| Separated | 4.4 | 2.45 | 7.88 | <0.001 |
| Never Married | 1.92 | 1.27 | 2.89 | 0.003 |
| Living with partner | 2.02 | 1.24 | 3.28 | 0.006 |

Unweighted sample size for regression is 3,055 participants (23 excluded who had missing values for one or more variables).

aOR, adjusted odds ratio; CI, confidence interval.
