# Supplementary material for: Trends in prevalence and treatment of antepartum and postpartum depression in the United States: Data from the national health and nutrition examination survey (NHANES) 2007 to 2018
Source: PLoS One. 2025 Apr 30;20(4):e0322536. doi: 10.1371/journal.pone.0322536 (PMC12043162; doi:10.1371/journal.pone.0322536)
Supplement: Attachment — Submitted filename: Response to Reviewers.docx [file pone.0322536.s006.docx]

1. Please ensure that your manuscript meets PLOS ONE's style requirements, including those for file naming. The PLOS ONE style templates can be found at

<https://journals.plos.org/plosone/s/file?id=wjVg/PLOSOne_formatting_sample_main_body.pdf> and

[https://journals.plos.org/plosone/s/file?id=ba62/PLOSOne_formatting_sample_title_authors_affiliations.pdf](https://track.editorialmanager.com/CL0/https:%2F%2Fjournals.plos.org%2Fplosone%2Fs%2Ffile%3Fid=ba62%2FPLOSOne_formatting_sample_title_authors_affiliations.pdf/1/010f0194e0e9bc5c-7b6a5579-4073-4c03-b58a-c60e59a5e956-000000/-PvkLGoWFVqA1UQiUypfE7CeDZve-Nxzz5MeEx47dxI=197)

**We have updated the manuscript to reflect PLOS ONE’s style requirements.**

2. Thank you for stating the following financial disclosure:

“Ms. Gardner's effort was supported by the T32 Training in Advanced Data and Analytics for Behavioral and Social Sciences Research (TADA-BSSR) training grant from the NIH National Heart, Lung, and Blood Institute (grant number 1T32HL1513232). “

Please state what role the funders took in the study.  If the funders had no role, please state: "The funders had no role in study design, data collection and analysis, decision to publish, or preparation of the manuscript."

If this statement is not correct you must amend it as needed.

Please include this amended Role of Funder statement in your cover letter; we will change the online submission form on your behalf.

**We have addressed this change in the cover letter.**

3. Thank you for stating the following in the Acknowledgments Section of your manuscript:

“The authors thank Dr. Kristin Sainani for editing the manuscript and critically evaluating the statistical analyses and conclusions.

Ms. Gardner's effort was supported by the T32 Training in Advanced Data and Analytics for Behavioral and Social Sciences Research (TADA-BSSR) training grant from the NIH National Heart, Lung, and Blood Institute (grant number 1T32HL1513232).”

We note that you have provided funding information that is not currently declared in your Funding Statement. However, funding information should not appear in the Acknowledgments section or other areas of your manuscript. We will only publish funding information present in the Funding Statement section of the online submission form.

Please remove any funding-related text from the manuscript and let us know how you would like to update your Funding Statement. Currently, your Funding Statement reads as follows:

“Ms. Gardner's effort was supported by the T32 Training in Advanced Data and Analytics for Behavioral and Social Sciences Research (TADA-BSSR) training grant from the NIH National Heart, Lung, and Blood Institute (grant number 1T32HL1513232).”

Please include your amended statements within your cover letter; we will change the online submission form on your behalf.

**We have removed the sentence about funding from the Acknowledgements’ section of the manuscript.**

4. Please review your reference list to ensure that it is complete and correct. If you have cited papers that have been retracted, please include the rationale for doing so in the manuscript text, or remove these references and replace them with relevant current references. Any changes to the reference list should be mentioned in the rebuttal letter that accompanies your revised manuscript. If you need to cite a retracted article, indicate the article’s retracted status in the References list and also include a citation and full reference for the retraction notice.

**The reference list is complete and correct. A single citation was added to include an updated reference to the literature regarding lifespan risk and prevention, with citations 55-57 renumbered accordingly. None of the cited papers have been retracted.**

Reviewers' comments:

Reviewer's Responses to Questions

**Comments to the Author**

1. Is the manuscript technically sound, and do the data support the conclusions?

The manuscript must describe a technically sound piece of scientific research with data that supports the conclusions. Experiments must have been conducted rigorously, with appropriate controls, replication, and sample sizes. The conclusions must be drawn appropriately based on the data presented.

Reviewer #1: Yes

Reviewer #2: Yes

2. Has the statistical analysis been performed appropriately and rigorously?

Reviewer #1: Yes

Reviewer #2: Yes

3. Have the authors made all data underlying the findings in their manuscript fully available?

The [PLOS Data policy](http://track.editorialmanager.com/CL0/http:%2F%2Fwww.plosone.org%2Fstatic%2Fpolicies.action%23sharing/1/010f0194e0e9bc5c-7b6a5579-4073-4c03-b58a-c60e59a5e956-000000/kmcZMDe9V2QcXkde9H3GUAC8_F9XwLHpZq9qRHHDA6o=197) requires authors to make all data underlying the findings described in their manuscript fully available without restriction, with rare exception (please refer to the Data Availability Statement in the manuscript PDF file). The data should be provided as part of the manuscript or its supporting information, or deposited to a public repository. For example, in addition to summary statistics, the data points behind means, medians and variance measures should be available. If there are restrictions on publicly sharing data—e.g. participant privacy or use of data from a third party—those must be specified.

Reviewer #1: Yes

Reviewer #2: Yes

4. Is the manuscript presented in an intelligible fashion and written in standard English?

PLOS ONE does not copyedit accepted manuscripts, so the language in submitted articles must be clear, correct, and unambiguous. Any typographical or grammatical errors should be corrected at revision, so please note any specific errors here.

Reviewer #1: Yes

Reviewer #2: Yes

5. Review Comments to the Author

Please use the space provided to explain your answers to the questions above. You may also include additional comments for the author, including concerns about dual publication, research ethics, or publication ethics. (Please upload your review as an attachment if it exceeds 20,000 characters)

Reviewer #1: Your group did a great job putting this paper together.

My recommendation for a minor revision is to change the way that you are using antidepressant-use as a definition for depression. Most of the antidepressants are also first-line treatments for anxiety, as you touched on in the discussion and limitations sections. It would be more accurate to split up the antidepressants by the corresponding ICD codes like you did in the discussion section and not include the patients with anxiety as their diagnosis.

**We agree that using ICD codes is preferred to only using the names of antidepressants; however, we had some limitations in our data. ICD codes for prescriptions were only included in NHANES data from 2013 onwards. When antidepressant use was defined using ICD-10 codes for depression available in 2013-2018, we observed lower depression prevalence in all three groups compared to the primary analysis, but we still found in our adjusted regression that postpartum women had lower odds of prevalent depression compared to the control group. Additionally, relationships between prevalent depression and participant characteristics, including educational attainment, income, race/ethnicity, and marital status were similar compared to the primary analysis. In the Discussion where we address defining antidepressant use broadly as a limitation, we have now included, “However, in a sensitivity analysis of years that included diagnosis codes for antidepressant prescriptions, our conclusions were similar compared to defining antidepressant use broadly.”**

Reviewer #2: Thank you to the authors for conducting this important research examining trends in antepartum and postpartum depression using NHANES data from 2007-2018. This well-designed study revealed lower depression prevalence in perinatal women compared to reproductive-age controls, with treatment rates lowest among antepartum women. The finding that mental health service utilization doubled for postpartum women in 2017-2018 suggests positive impacts of the 2016 USPSTF screening guidelines. The study's nationally representative sample and rigorous methodology provide valuable insights for improving perinatal mental healthcare delivery. Thank you for your effort in advancing this area of research. You can see my comments below:

•Abstract:
1.In the Methods (lines 12-13), the authors mentioned: “Multivariable logistic regression adjusted for age, insurance, race/ethnicity, education, and marital status estimated odds ratios and 95% confidence intervals.” I noticed that low-income status, mentioned in lines 137-138, was not included as a covariate in the multivariable logistic regression analysis. Given the well-established relationship between socioeconomic status and depression, it would be helpful if the authors could explain their rationale for excluding this variable from the adjusted models. Was this decision based on sample size considerations, degrees of freedom limitations, potential collinearity with other socioeconomic indicators like education and insurance status, or other methodological considerations?

**Ideally, we would have liked to include a more precise measurement of income (in our data, this would be ratio of family income to poverty level) as a covariate in our multivariable regression; however, 460 participants were missing data for this variable. For example, 8.3% of the antepartum group was missing information for this variable, and since this group was already small (n=314), we did not want to sacrifice sample size. Since health insurance type is a well-known proxy for income status, we chose to use health insurance in our adjusted regression since only 8 participants were missing this variable. For reference, Table 1 indicates the proportion of missingness for each descriptive characteristic, including ratio of family income to poverty level and health insurance type. We have added the following to the Limitations’ section of the Discussion: “Given the well-established link between socioeconomic status and depression, we initially planned to adjust for the income-to-poverty ratio from NHANES data. However, because this variable had a relatively high rate of missingness, we used insurance type as a proxy instead.”**

•Introduction:
2. In lines 62-63, the authors mentioned: “However, these findings are derived from analyses of commercial claims databases, which notably exclude mothers with public insurance.” Given that the exclusion of mothers with public insurance in previous commercial claims databases was presented as one of the key rationales for conducting this study, I recommend expanding the discussion section to address the significance of including publicly insured women in this analysis. This would strengthen the manuscript by explicitly highlighting how the findings from this more representative sample, including both publicly and privately insured women, contribute to our understanding of perinatal depression prevalence and treatment patterns across different insurance status groups.

**This is an excellent suggestion; we have incorporated the following sentence in our Conclusion: “This is the first study to evaluate antepartum and postpartum depression trends in a cohort of women with private and public insurance, increasing the generalizability of our findings.”**

•Methods:
3. Regarding the study groups and inclusion criteria, I suggest providing clarification regarding the handling of participants' prior history of depression or other mental disorders. If this information is available in NHANES, it would be valuable to either exclude these individuals in a sensitivity analysis or at minimum flag and report them separately. Please clarify if such data is available and explain the rationale for the current approach.

**We agree that history of mental health conditions is an important confounding factor in determining the association between perinatal status and prevalent depression. Unfortunately, NHANES does not collect information on prior history of depression or other mental disorders for the years analyzed (2007-2018). We include this in our limitations’ section of the Discussion: “…key confounding factors, such as a history of mental health conditions…that increase depression risk, were not collected.”**

4. Please refer to my earlier comment (comment #1) regarding the multivariable logistic regression model specified in lines 152-153, which addresses the variables included in the adjusted analysis.

**We have addressed this comment above.**

Results:
5. Could the authors provide their rationale for including Language and BMI in Table 1? Understanding the relevance of these variables to perinatal depression prevalence and treatment would help readers better interpret their inclusion in the baseline characteristics.

**We evaluated Language and BMI as characteristics potentially related to prevalent depression, and from Table 3 Spanish language appears to be associated with lower prevalent depression, while higher BMI is associated with higher rates of prevalent depression. While we did not choose to focus on these findings, we present these variables in Tables 1 and 3 to readers who may be interested in exploring these associations in future research as well as for transparency.**

Discussion:
6. The statement in lines 336-337 "pregnancy and postpartum may confer protective benefits against depressive symptoms for some women" warrants more careful interpretation. While the data shows lower depression prevalence in perinatal women compared to controls, suggesting a protective effect may imply causation from cross-sectional data. Consider rephrasing to "Our findings indicate lower prevalence of depressive symptoms among pregnant and postpartum women compared to non-pregnant controls"

**We have rephrased this statement as suggested, "Our findings indicate lower prevalence of depressive symptoms among pregnant and postpartum women compared to non-pregnant controls."**

7. I would like to thank the authors for conducting this comprehensive and insightful research. The supplemental documents were particularly helpful in addressing potential methodological questions that I had and providing additional context for the findings.
